# Assessment of bacterial proteome remodeling under virulence-inducing conditions for molecular pharming applications

**DOI:** 10.1128/mra.01139-24

**Published:** 2025-10-17

**Authors:** Kiyan Kheradvar, Nicholas Prudhomme, Michael D. McLean, Doug Cossar, Jennifer Geddes-McAlister

**Affiliations:** 1 Molecular and Cellular Biology Department, University of Guelph 3653, Guelph, Canada; 2 PlantForm Corp. Canada, Toronto, Ontario, Canada; Indiana University Bloomington, Bloomington, Indiana, USA

**Keywords:** molecular pharming, *Agrobacterium*, virulence, proteomics

## Abstract

Using mass spectrometry-based proteomics, we identified virulence proteins from *Agrobacterium tumefaciens* with significantly increased abundance following exposure to chemical (acetosyringone) and biological (leaf extract from *Nicotiana benthamiana*) inducers. Leaf extracts could be a cost-effective alternative to acetosyringone for inducing pathogen virulence for the molecular pharming industry.

## ANNOUNCEMENT

Molecular pharming is the process of producing biologics (e.g., antibodies, vaccines) using the natural infection process between the plant bacterial pathogen, *Agrobacterium tumefaciens,* and the host, *Nicotiana benthamiana* (1, 2). The technology supports increased scalability, enhanced safety, lower production costs, and proper molecular modifications compared with cell production systems (3). During the infection process, *A. tumefaciens* delivers transfer (T)-DNA from its tumor-inducing (Ti) plasmid through the type-IV secretion system (T4SS) into the plant cell for production of specific targets (4–6). In this study, we measured changes in the bacterial proteome under chemical and biological conditions known to influence infectivity (7).

Plant leaves were harvested from *N. benthamiana* (KDFX plant line derived from USDA accession “TW16”), sterilized (i.e., 30 s wash in 70% ethanol, 10 s wash in sterile Milli-Q [MQ] water, 10 min wash in 0.6% active bleach, and two 10 s washes in sterile MQ water), and dried aseptically prior to flash freezing and storage at −80 °C. *A. tumefaciens* (strain At564A, derived from C58, containing the Trastuzumab vector pFC0058) was grown in quadruplicate in Luria-Bertani broth with kanamycin (50 µg/mL) and rifampin (25 µg/) overnight at 28 °C with shaking (200 rpm) in the dark and sub-cultured to a normalized OD_600nm_ of 0.2, as we previously described (8, 9). Bacterial virulence was induced at room temperature for 24 h in the dark by: 100 µM acetosyringone (TCI Chemicals) in agroinfiltration medium , 40 mg/mL plant leaves blended in agroinfiltration medium, or supernatant of 40 mg/mL segmented plant leaves pre-incubated with agroinfiltration medium for 48 h at room temperature (referred to as leaf extract). An uninduced control contained only agroinfiltration medium. Notably, the acetosyringone and untreated control experiment was performed independently from the biological (leaf extract and blended leaf) experiments.

) overnight at 28°C with shaking (200 rpm) in the dark and sub-cultured to a normalized OD_600nm_ of 0.2, as we previously described (8, 9). Bacterial virulence was induced at room temperature for 24 h in the dark by: 100 µM acetosyringone (TCI Chemicals) in agroinfiltration medium , 40 mg/mL plant leaves blended in agroinfiltration medium, or supernatant of 40 mg/mL segmented plant leaves pre-incubated with agroinfiltration medium for 48 h at RT (referred to as leaf extract). An uninduced control contained only agroinfiltration medium. Notably, the acetosyringone and untreated control experiment was performed independently from the biological (leaf extract and blended leaf) experiments.

Cell pellets were collected at 4,696×*g* for 10 min, washed twice with phosphate-buffered saline (PBS), and processed as previously described (9, 10). Briefly, pellets were resuspended in 100 mM Tris-HCl (pH 8.5) containing a proteinase inhibitor cocktail tablet, lysed in 2% sodium dodecyl sulfate (SDS) via probe sonication on ice bath (30% amplitude, 3 cycles of 30 s on/30 s off). After the addition of dithiothreitol (DTT; 10 mM final concentration), samples were incubated at 95 °C for 10 min at 800 rpm, cooled, and iodoacetamide (IAA; 55 mM final concentration) was added, followed by incubation in the dark at RT for 20 min. Proteins were precipitated overnight using acetone (80% final volume) at −20 °C, centrifuged at 16,100×*g*, washed twice with 80% acetone, air-dried, resolubilized in 8 M Urea/40 mM HEPES, and quantified by tryptophan release (11). Samples (25 µg) were digested overnight at 37 °C with trypsin/LysC (protein:enzyme 50:1 w/w) and purified on STAEG-tips (12). Dried peptides were quantified at OD_205nm_ and 3 µg of peptides was resuspended in 0.1% (v/v) trifluoroacetic acid, separated by liquid chromatography (RSLC EasyNano, Thermo Fisher) and electrospray ionization (50 cm PepMap column of 75 µm and C18 resin) coupled to an Orbitrap Exploris 240 (Thermo Fisher) mass spectrometer operated in data dependent-acquisition mode (MS1 scan for top10 peptides, MS2 scan for peptide sequencing, m/z range 400–1,600, resolution 60,000). Peptides were analyzed using a 180 min gradient for chemical conditions (acetosyringone) as the positive control; peptides were analyzed using a 90 min gradient for biological conditions (leaf extract and blended leaves) for instrument time optimization.

Mass spectrometry data were analyzed using MaxQuant (v. 2.1.0.0) and Perseus (v. 2.0.7.0) software (13, 14). The reference sequence of *A. tumefaciens* (16 December 2019; 5,344 proteins) supplemented with Trastuzumab heavy chain, light chain, two P19 sequences, and neomycin phosphotransferase II (15) was used for searching. Default parameters were set in MaxQuant with label-free quantification (minimum ratio count of 2), minimum peptides of 2, and match between runs enabled. In Perseus, filtering for modified by site (e.g., post-translational modification), potential contaminants, and reverse peptides was performed and proteins present in three of four replicates within one group were retained. NaN values were imputed from a normal distribution (width 0.3, down-shift 1.8), and “Majority protein IDs” were annotated using the UniprotKB database (16). Volcano plots evaluated significant changes in protein abundance by Student’s *t*-test, *P* < 0.05, Benjamini-Hochberg (17) false discovery rate = 0.05, S_0_ = 1.

**Fig 1:**
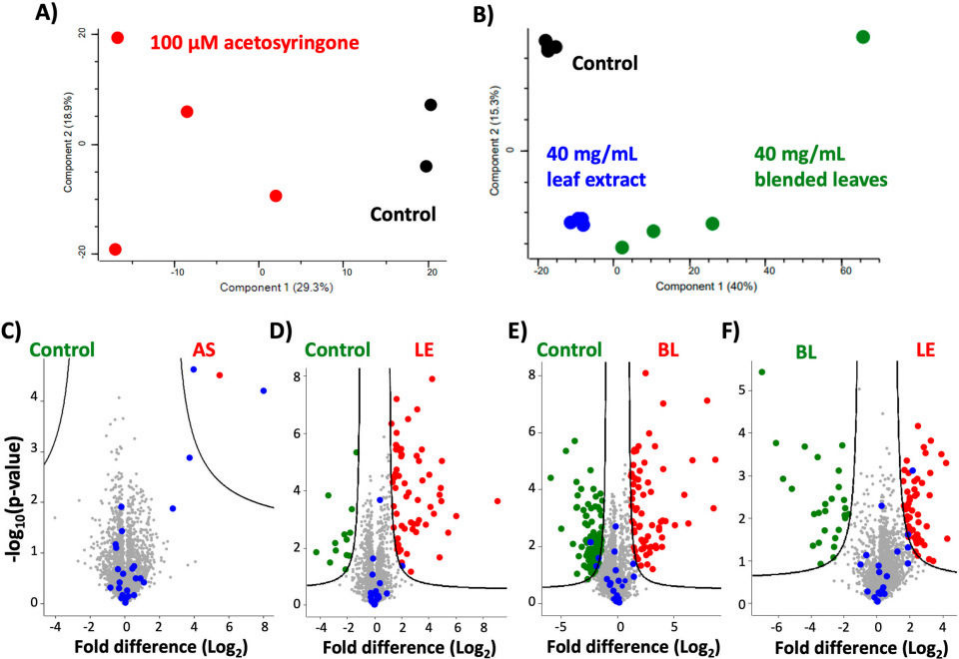
Proteome profiling of *A. tumefaciens* under chemical and biological induction of virulence. (**A**) Principal component analysis of control (agroinfiltration medium) and chemical (acetosyringone) conditions. (**B**) Principal component analysis of control (agroinfiltration medium) and biological (leaf extract and blended leaves) conditions. (**C**) Volcano plot for control (agroinfiltration medium) and chemical (AS; acetosyringone) conditions. (**D**) Volcano plot for control (agroinfiltration medium) and biological (LE; leaf extract) conditions. (**E**) Volcano plot for control (agroinfiltration medium) and biological (BL; blended leaves) conditions. (**F**) Volcano plot for leaf extract (LE) and blended leaves (BL). Blue = known virulence-associated proteins. For volcano plots, Student’s *t*-test *P*-value < 0.05, FDR = 0.05, S_0_ = 1. Performed in biological quadruplicate.

We observed clear separation between the controls and chemical (acetosyringone) (Fig. 1A) or biological (leaf extract and blended leaves) (Fig. 1B) treated samples. Only three significantly different proteins were defined by chemical treatment (Fig. 1C) compared with 76 proteins significantly different between the control and leaf extract (Fig. 1D), and 174 proteins significantly different between control and blended leaves (Fig. 1E). Importantly, virulence-associated bacterial proteins (e.g., VirH1, VirH2, T4SS) (2) were significantly increased under chemical treatment (acetosyringone), biological treatment (leaf extract), and upon comparison between leaf treatments (Fig. 1E). Overall, this study demonstrates proteome remodeling of *A. tumefaciens* in response to chemical and biological treatments, but limited insight into regulation of virulence factor production is evident.

## Data Availability

The mass spectrometry-based proteomics data is available through PRIDE Proteome Exchange with accession number PXD047349.
